# Gastrointestinal bleeding among oral anticoagulant users: a comprehensive 7-year retrospective review using Türkiye’s national health data system

**DOI:** 10.55730/1300-0144.5879

**Published:** 2024-07-10

**Authors:** Nuray YILMAZ ÇAKMAK, Naim ATA, Serdar Can GÜVEN, Emin GEMCİOĞLU, Murat ÇAĞLAYAN, Mahir ÜLGÜ, Şuayip BİRİNCİ

**Affiliations:** 1 Department of Internal Medicine Sciences, Faculty of Medicine, Yıldırım Beyazıt University, Ankara, Turkiye; 2 Department of Strategy Development, Republic of Türkiye Ministry of Health, Ankara, Turkiye; 3 Division of Rheumatology, Department of Internal Medicine Sciences, Ankara Bilkent City Hospital, Ankara, Turkiye; 4 Department of Internal Medicine Sciences, Faculty of Medicine, Health Sciences University, Ankara, Turkiye; 5 Department of Microbiology Sciences, Faculty of Medicine, Health Sciences University, Ankara, Turkiye; 6 Republic of Türkiye Ministry of Health, General Directorate of Health Information System, Ankara, Turkiye; 7 Republic of Türkiye Ministry of Health, Ankara, Turkiye

**Keywords:** Direct oral anticoagulants, gastrointestinal bleeding, vitamin K antagonists, warfarin, big data

## Abstract

**Background/aim:**

The comparative risk of gastrointestinal bleeding (GIB) among users of direct-acting oral anticoagulants (DOACs) versus vitamin K antagonists (VKAs) is a topic of ongoing debate. This study leverages a comprehensive national health database to evaluate the incidence of GIB, associated risk factors, and postbleeding management strategies among anticoagulated patients.

**Materials and methods:**

Utilizing the Turkish Ministry of Health’s e-Nabız system, we conducted a retrospective analysis of patients treated with DOACs and warfarin from January 2017 to July 2023. GIB events were identified using ICD codes, and comorbidities, prior medication use, interventions, and mortality rates were analyzed. Drug survival and patterns of changes following GIB were also evaluated.

**Results:**

Among 102,545 patients with a GIB event during anticoagulant treatment, DOAC users were older with a higher prevalence of comorbidities, except for chronic obstructive lung disease, compared to VKA users. GIB-related mortality was 0.6% in the DOAC group and 0.4% in the VKA group at admission after the GIB (p < 0.01). In all drug groups, approximately half of the patients discontinued anticoagulation due to GIB after 3 months, the rate being highest with apixaban (61.9%). In patients who continued anticoagulation, the anticoagulant prior to GIB remained the most common agent in all groups, with rivaroxaban having the highest retention rate (40.7%).

**Conclusion:**

This nationwide study indicates a higher frequency of GIB in DOAC users versus VKA users, with age and comorbidities potentially contributing to this trend. Mortality rates were comparable to the previous literature but warrant further investigation. The significant rate of discontinuation following GIB raises concerns about ongoing anticoagulation management. These findings underscore the need for cautious case management.

## 1. Introduction

Oral anticoagulants (OACs) are widely used agents for the treatment and prevention of thrombotic events. Vitamin K antagonists (VKAs) were previously the cornerstone of OAC treatment, but direct-acting OACs (DOACs) have provided an alternative in recent years with benefits like no need for routine international normalized ratio (INR) follow-up and short time of action in scenarios like emergency surgery or bleeding [1].

Rivaroxaban was the pioneer of DOACs, launched in 2008 and followed by apixaban, dabigatran, and edoxaban in the following years [2]. Fewer drug interactions, short time of action, and shorter half-life have made DOACs preferable to VKAs [3]. However, concerns regarding the increased risk of gastrointestinal bleeding (GIB) in comparison to VKAs arose as a setback for DOACs [4]. The knowledge in the literature regarding bleeding risk for users of DOACs is controversial since the findings are contradictory [5–8]. Furthermore, various other factors could be related to GIB such as age, comorbidities, the use of drugs that irritate the gastrointestinal tract, and drug interactions [9,10]. Therefore, it is still to be fully elucidated whether there is a major difference in the risk for GIB between VKAs and DOACs.

The Turkish Ministry of Health established a database system, the e-Nabız system, in 2015 where the health data of individuals such as demographics, diagnoses in terms of International Classification of Diseases (ICD) codes, treatments, and imaging, laboratory, and pathology results are recorded for the entire population [11]. In addition, the system has also been integrated into big data analysis technologies as of 2016, providing valuable results for the country’s healthcare status and enabling research with the permission of the Ministry of Health.

In this study, using the aforementioned big data platform of the Ministry of Health, we retrospectively investigated GIB occurrence in VKA and DOAC users and evaluated postbleeding drug retention rates and potential risk factors that can affect GIB risk [10].

## 2. Materials and methods

This study was conducted as a retrospective, nationwide, big data study. The records of individuals who were prescribed DOACs and warfarin between January 2017 and July 2023 were investigated for the presence of codes for apixaban, edoxaban, rivaroxaban, dabigatran, and warfarin (SGKFXF, SGKFZM, SGKG0H, SGKFOH, SGKFWS, YDIA5U, YDIA5V, SGKFV1) using the aforementioned national database with the permission of the Ministry of Health [11]. From among these patients, cases of GIB during OAC treatment were detected by using ICD codes K92.0, K92.2, and K92.1. Indications for OACs were screened using the ICD codes for atrial fibrillation (I48), venous thromboembolism (I82), and heart valve replacement surgery (Z95.4, Z95.2). Likewise, comorbid diseases were screened via ICD codes, including hypertension (I10–I15), diabetes mellitus (E10–E14), cerebrovascular diseases (I60–I69), chronic obstructive pulmonary disease (J42–J45), heart failure (I50), and chronic kidney disease (N18).

Prior proton pump inhibitor (PPI) and H2 blocker use, concomitant use of antiaggregants (acetylsalicylic acid, clopidogrel, ticagrelor, prasugrel) and other anticoagulants or drugs irritating the gastrointestinal tract before the GIB event within last month, interventions for GIB, transfusions and treatment for GIB, mortality related to GIB (mortality during hospitalization for GIB), and changes in OACs among GIB survivors were recorded. Patients who did not have a new anticoagulant prescription within 3 months after bleeding were defined as patients who did not continue taking medication. Patients with multiple medication switches after GIB were excluded.

Statistical analyses were conducted using IBM SPSS Statistics 27 (IBM Corp., Armonk, NY, USA). Variables were evaluated visually (plots and histograms) and by Kolmogorov–Smirnov test. Continuous variables were presented as either mean ± standard deviation (SD) or median (min–max). Categorical variables were presented as numbers and percentages. Continuous variables were compared by either the Mann–Whitney U test or Student t-test according to normality. Categorical variables were compared by chi-square test. Values of p < 0.05 were accepted as significant.

The study was conducted following the principles of the Declaration of Helsinki and approved by the Turkish Ministry of Health with a waiver of informed consent for retrospective data analysis.

## 3. Results

**Figure 1:**
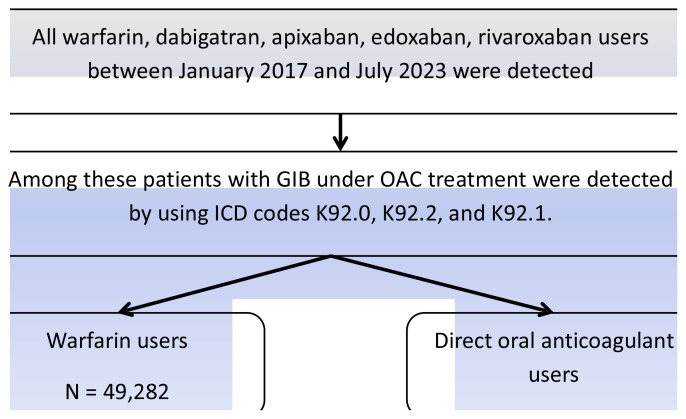
Patient inclusion flow chart.

A total of 102,545 patients who had experienced a GIB event during OAC or warfarin usage were included in the study (Figure 1). A total of 53,263 DOAC users had a GIB event and 49,282 VKA users had a GIB event. Demographics, comorbidities, and numbers of DOAC and VKA users are presented in Table 1. In the DOAC group, the mean age was 73.82 ± 10.91 years, while it was 69.37 ± 12.99 in the VKA group (p < 0.01). While 56.5% of DOAC users were female, 51.4% of VKA users were female (p < 0.01, odds ratio (OR) ± 95%: 0.836). The most common comorbidity was hypertension and the most common indication for OAC was atrial fibrillation in both groups (DOAC: n = 48,150 (90.5%), VKA: n = 40,900 (83.0%), p = 0.141). All comorbidities were detected to be more frequent in DOAC users. Chronic obstructive lung disease was also detected (DOAC: n = 26,581 (50.0%), VKA: n = 13,196 (26.8%), p = 0.197).

**Figure 2:**
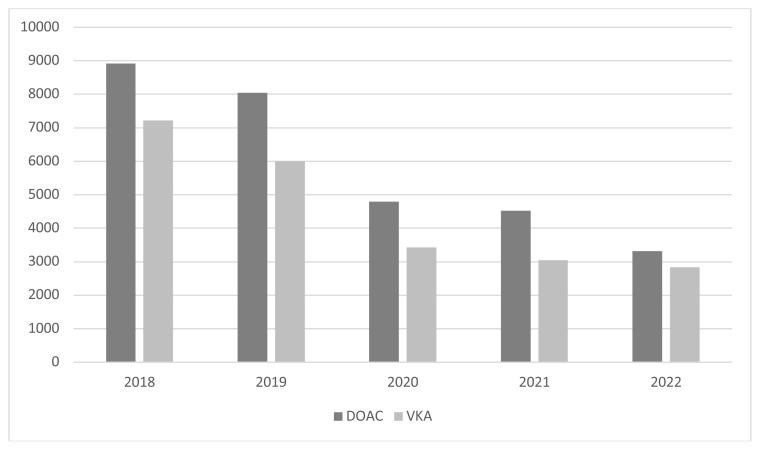
Number of annual GIB events in DOAC and VKA users between the years of 2018 and 2022.

The annual numbers of GIB events were higher every year in the DOAC group (Figure 2). Frequencies of PPI, H2 blocker, antiaggregant, other anticoagulant, and nonsteroidal antiinflammatory drug (NSAID) use before GIB are presented in Table 2. When interventions for GIB were evaluated, it was found that endoscopy was applied for 35.9% of DOAC users and 38.1% of VKA users. Fresh frozen plasma was administered to 13.5% of DOAC users and 25.2% of VKA users (p < 0.01). Interventions for the treatment of GIB are given in Table 3. GIB-related mortality was 0.6% in the DOAC group and 0.4% in the VKA group after hospital admission following GIB (p < 0.01). Rates of drug changes and discontinuation are presented in Table 4. In all drug groups, approximately half of the patients discontinued anticoagulation due to GIB in a 3-month period, and this rate was highest for apixaban (61.9%). In patients who continued anticoagulation, the anticoagulant prior to GIB remained the most common agent in all groups, with rivaroxaban having the highest retention rate (40.7%). In DOAC users who switched to another drug, another DOAC was most commonly selected. In VKA users, in the event of a switch, rivaroxaban was the most frequently selected DOAC (12.6%).

## 4. Discussion

Our study presents nationwide data regarding GIB among DOAC and VKA users over a period of 7 years. The annual number of patients with GIB was higher among the DOAC users and DOAC users were slightly older. Comorbid conditions were more frequent in the DOAC group, except for chronic kidney disease. Concomitant use of gastroprotective agents and drugs irritating the gastrointestinal tract were similar between the groups. GIB-related mortality was also similar. A significant portion of the patients discontinued anticoagulation after the GIB event.

The complication of GIB is a major concern in patients receiving anticoagulation treatment. Since DOACs were first introduced, it has been a matter of concern whether they are safer or riskier in terms of GIB compared to VKA. The cumulative data in the literature regarding this issue suggest that there is no difference in GIB risk, but the severity of bleeding may be a concern in VKA users [12]. In our study, total and annual events were more frequent in the DOAC group. The mortality rate was similar between the two groups. We assume that GIB event rates may have been reduced annually due to the possibility of patients with potential bleeding risk experiencing a GIB event and discontinuing the drug. Rates of nonbleeders and drug retention may have increased for this reason.

Increased age and comorbidities in patients receiving anticoagulation treatment are risk factors for GIB [13]. A mean age of 80 years was previously reported for patients with GIB events during anticoagulation treatment, but no significant difference was found between the DOAC and VKA groups [9]. In our study, the mean age was older and comorbid conditions were more frequent among DOAC users. It can be speculated that due to less drug interaction and no need for INR follow-up, DOACs may have been preferred more frequently in the elderly population and this, in addition to more frequent comorbidities, may be a reason for more GIB events in the DOAC group.

Since DOACs began to be used in clinical practice, bleeding side effects have always been at the forefront. Although some studies claim that they cause less bleeding, other studies show that the risk of bleeding is the same as that reported for VKAs [14,15]. When evaluated in terms of drug effects or differences between drugs, some studies have associated both dabigatran and rivaroxaban with a significantly increased risk of gastrointestinal bleeding with a standardized 1-year risk compared to apixaban [16]. Since our study was retrospective and we did not have information about the bleeding risks of the patients, it would be inappropriate to make a definitive judgment, but caution should be exercised in terms of the possibility of GIB during DOAC treatment. More careful use of DOACs may be necessary, especially in patients with high bleeding risk.

According to guidelines published in 2012, the use of DOACs in cases of nonvalvular atrial fibrillation has been accepted in daily practice [17]. In our study, we found that the most common indication for starting medication in both the DOAC and VKA groups was nonvalvular atrial fibrillation.

Comorbidities are known to increase the risk of gastrointestinal bleeding. In our study, the most common comorbidity was hypertension in both groups [18]. When compared in terms of bleeding percentages, more GIB events were observed in the DOAC groups in our study. This finding contradicts the literature. It is accepted in the literature that one of the risk-increasing factors for GIB is the use of NSAIDs. In our study, however, we saw similar drug use in both groups [19].

Concomitant drug use is an important factor in the development of GIB in patients undergoing anticoagulant treatment, and 75.39% of DOAC users and 70.68% of VKA users had been prescribed a PPI within the last month due to a GIB event. The frequency of other drugs associated with GIB was similar between the groups except for low-molecular-weight heparin, which was more frequent in the VKA group. This was probably due to the need for bridging when VKAs are first administered. When interventions for GIB were compared, fresh frozen plasma was more frequently administered to VKA users [20].

In most cases, the indication for anticoagulation treatment persists after GIB. Retention of the same drug versus a switch to another oral anticoagulant is still a matter of debate [18]. Dawwas et al. reported low bleeding rates with apixaban in comparison to rivaroxaban [21], similar to the result of the study conducted by Rodilla et al. [22]. Interestingly, in our study rivaroxaban had the highest retention rate and it was the most preferred agent in the event of a switch. Another important result of our study is that nearly 50% of the patients did not continue oral anticoagulation treatment. Unfortunately, our data cannot elucidate the true reason for that. Several factors could be underlying this finding, such as patient concerns, physician concerns, and decisions to switch to parenteral anticoagulants [23].

Mortality is the most important outcome of GIB. Several retrospective studies reported rates of 2% and 10% mortality in OAC users [9,24]. Our nationwide findings suggest rates of 7.1% in DOAC and 6.2% in VKA users. Our study could not identify the reason for these relatively high mortality rates. This is a matter of concern and should be further investigated.

There are several limitations of this study. It had a retrospective and cross-sectional design. Furthermore, the data were obtained through ICD codes and clinical assessment was not incorporated, particularly regarding the severity of bleeding, bleeding location in the gastrointestinal system, and the period between the first symptom and presentation to a healthcare institution. Furthermore, our study could evaluate only the variables recorded within e-Nabız and there are numerous other confounding factors that could not be evaluated. Finally, a limitation of data integration is that it can be challenging to combine data from multiple sources due to the need for a standardized format and structure, which may not always be possible or feasible to achieve. However, this setback is seen in all big data studies conducted using national healthcare databases and is acceptable since the magnitude of the data compensates for potential stochastic errors in data integration.

We defined individuals who had no drug prescription for 3 months as the group that did not continue taking medication after bleeding. This is another limitation of the study because patients could receive medication during the COVID-19 pandemic without the need for a prescription, and patients with medication at home may not have appeared to have taken medication according to the system even if they were using it again. Unfortunately, this was a limitation that could not be avoided.

In conclusion, the results of this large nationwide study revealed that GIB was more frequent among DOAC users in comparison to VKA users. Other contributing factors could not be precisely identified. Furthermore, a higher mortality rate was observed compared to the literature, which requires further investigation of the potential causes. Another point of concern is that nearly half of the patients did not continue using oral anticoagulation drugs. Despite the limitations, this study has highlighted important aspects and future research in Türkiye can consider these points.

## Figures and Tables

**Table 1:** Demographics, comorbidities, and reasons for anticoagulation treatment in patients with a GIB event.

	DOAC users	VKA users	
	n = 53,263	n = 49,282	p
Age, years, mean ± SD	73.82 ± 10.9	69.37 ± 12.99	<0.01
Sex, female, n (%)	30,040 (56.5)	25,338 (51.4)	0.25
Reason for anticoagulation, n (%)			
Atrial fibrillation	48,150 (90.5)	40,900 (83.0)	<0.01
Venous thromboembolism	19,380 (27.0)	15,200 (30.8)	<0.01
Hearth valve replacement	0 (0)	20,955 (41.6)	0
Comorbidities, n (%)			
Diabetes mellitus	23,098 (43.4)	19,104 (38.8)	0.825
Hypertension	49,354 (92.8)	43,806 (88.9)	<0.01
Cerebrovascular event	18,544 (34.9)	14,095 (28.6)	<0.01
Coronary artery disease	10,894 (20.5)	9471 (19.2)	<0.01
Chronic obstructive pulmonary disease	26,581 (50.0)	13,196 (26.8)	0.197
Chronic kidney disease	6019 (11.3)	6394 (13.0)	<0.01
Chronic liver disease	22,066 (41.5)	18,699 (37.9)	<0.01
Malignancy	6499 (12.2)	4780 (9.7)	<0.01

**Table 2:** Concomitant use of other drugs associated with GIB prior to the GIB event.

	DOAC users	VKA users	p
	n = 53,263	n = 49,282	
Proton pump inhibitors, n (%)	16,019 (75.4)	14,541 (70.7)	<0.01
H2 receptor blockers, n (%)	667 (3.1)	605 (2.9)	0.034
Nonsteroidal antiinflammatory drugs, n (%)	5044 (23.7)	5041 (24.5)	0.049
Acetylsalicylic acid, n (%)	3437 (16.2)	3778 (18.4)	<0.01
Heparin, n (%)	3 (0.01)	8 (0.04)	0.041
Low-molecular-weight heparin, n (%)	1677 (7.9)	3667 (17.8)	<0.01
Ticagrelor, n (%)	46 (0.2)	39 (0.2)	0.771
Fondaparinux, n (%)	3 (0.01)	3 (0.01)	<0.01
Prasugrel, n (%)	1419 (6.6)	1319 (6.4)	<0.01

**Table 3:** Interventions for the treatment of GIB.

	DOAC users	VKA users	
	n = 53,263	n = 49,282	p
Gastroscopy, n (%)	19,098 (35.9)	18,783 (38.11)	0.001
Colonoscopy, n (%)	11,070 (20.8)	9852 (20.0)	<0.001
Thrombocyte suspension, n (%)	3032 (5.3)	3405 (6.9)	<0.001
Fresh frozen plasma, n (%)	7191 (13.5)	12,431 (25.2)	<0.001
Cryoprecipitate, n (%)	248 (0.5)	356 (0.7)	<0.001
Erythrocyte suspension, n (%)	32,184 (60.5)	32,178 (65.3)	<0.001
Tranexamic acid, n (%)	621 (1.2)	630 (1.3)	0.107

**Table 4:** Anticoagulant discontinuation and changes of drugs after GIB in nonvalvular atrial fibrillation and venous thromboembolism.

	Drug after GIB, n (%)					
Drug before GIB	VKA	Apixaban	Dabigatran	Edoxaban	Rivaroxaban	Anticoagulant discontinuation
VKA n = 49,282	7820 22.4%	2158 6.2%	417 1.2%	1546 4.4%	4402 12.6%	18,620 53.3%
Dabigatran n = 6450	115 1.8%	542 8.4%	1659 25.7%	319 4.9%	592 9.2%	3223 50.0%
Rivaroxaban n = 24,815	590 2.4%	1032 4.2%	151 0.6%	613 2.5%	10,104 40.7%	12,325 49.7%
Apixaban n = 10,526	247 2.3%	2870 27.3%	63 0.6%	278 2.6%	500 4.8%	6568 62.4%
Edoxaban n = 4758	132 2.8%	153 3.2%	10 0.2%	1913 40.2%	215 4.5%	2335 49.1%
